# LncRNA GAS5 Regulates Myometrial Cell Contractions in an m6A-Dependent Manner

**DOI:** 10.1093/function/zqaf009

**Published:** 2025-03-07

**Authors:** Yue Sun, Min Zhang, Tianjun Wang, Shiyun Huang, Qing Zuo, Lanhua Liu, Runrun Feng, Yufei Han, Cen Cao, Haiyan Sun, Yihan Lu, Xinxin Zhu, Yuping Tang, Shuang Wu, Guoqiang Ping, Lizhou Sun, Zhiping Ge, Ziyan Jiang

**Affiliations:** Department of Obstetrics, First Affiliated Hospital of Nanjing Medical University, Nanjing 210029, Jiangsu, China; Department of Obstetrics, First Affiliated Hospital of Nanjing Medical University, Nanjing 210029, Jiangsu, China; Department of Obstetrics, First Affiliated Hospital of Nanjing Medical University, Nanjing 210029, Jiangsu, China; Department of Obstetrics, First Affiliated Hospital of Nanjing Medical University, Nanjing 210029, Jiangsu, China; Department of Obstetrics, First Affiliated Hospital of Nanjing Medical University, Nanjing 210029, Jiangsu, China; Department of Obstetrics, People’s Hospital of Taixing, Taizhou 225400, Jiangsu, China; Department of Obstetrics, First Affiliated Hospital of Nanjing Medical University, Nanjing 210029, Jiangsu, China; Department of Obstetrics, First Affiliated Hospital of Nanjing Medical University, Nanjing 210029, Jiangsu, China; Department of Obstetrics, First Affiliated Hospital of Nanjing Medical University, Nanjing 210029, Jiangsu, China; Department of Obstetrics, First Affiliated Hospital of Nanjing Medical University, Nanjing 210029, Jiangsu, China; Department of Obstetrics, First Affiliated Hospital of Nanjing Medical University, Nanjing 210029, Jiangsu, China; Department of Obstetrics, First Affiliated Hospital of Nanjing Medical University, Nanjing 210029, Jiangsu, China; Department of Obstetrics, Dongtai People’s Hospital, Yancheng 224000, Jiangsu, China; Department of Obstetrics, Baoying People’s Hospital, Yangzhou 225000, Jiangsu, China; Department of Pathology, First Affiliated Hospital of Nanjing Medical University, Nanjing 210029, Jiangsu, China; Department of Obstetrics, First Affiliated Hospital of Nanjing Medical University, Nanjing 210029, Jiangsu, China; Department of Obstetrics, First Affiliated Hospital of Nanjing Medical University, Nanjing 210029, Jiangsu, China; Department of Obstetrics, First Affiliated Hospital of Nanjing Medical University, Nanjing 210029, Jiangsu, China; Department of Obstetrics, People’s Hospital of Taixing, Taizhou 225400, Jiangsu, China

**Keywords:** labor onset, lncRNA GAS5, METTL3, IGF2BP1, TPM4, N6-methyladenosine (m6A)

## Abstract

LncRNAs are engaged in signaling pathways in human physiological and pathological states. However, LncRNAs mediate the onset of human labor still remains unknown. RNA sequencing of lower segment myometrium (in labor vs. not in labor) was analyzed. N6-Methyladenosine (m6A) complexes were detected by RIP and meRIP in human myometrial cells. Plasmid and siRNA transfection was performed, and contraction ability was assessed. RNA pulldown, silver staining, protein mass spectrometry, and RIP were used to identify binding proteins. FISH and immunofluorescence costaining were applied to assess the coexpression. GAS5 was upregulated in human myometrium after labor onset. METTL3 and IGF2BP1 maintained GAS5 RNA stability based on actinomycin assay, thus strengthening the contraction of myometrial cells. RIP and meRIP revealed the binding sites of GAS5 with METTL3 and IGF2BP1, respectively. Furthermore, GAS5 binds TPM4 in cytoplasm of myometrium cells and transports TPM4 to the contraction filaments. m6A RNA modifications were also noted in the mouse myometrium after labor onset. These findings highlighted the critical role of m6A modification in GAS5, providing a new method to explore RNA epigenetic regulatory patterns in human parturition.

## Graphical Abstract

**Graphical Abstract:**
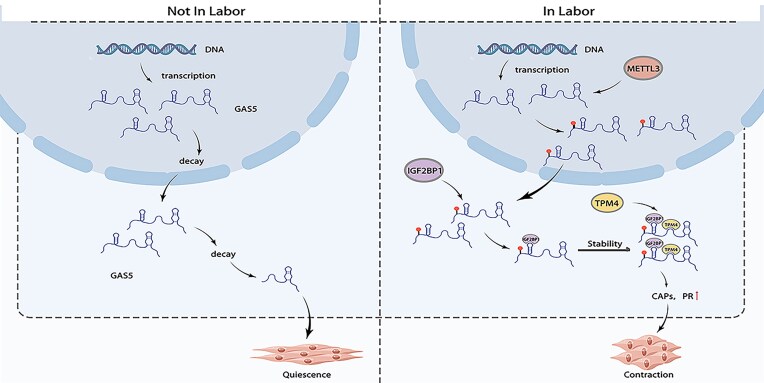
LncRNA GAS5 Regulates Myometrial Cell Contractions

## Introduction

The mechanisms involved in human parturition are highly complex and involve the mother, fetus, and placenta. The uterus undergoes extensive structural remodeling and remains quiescent over the course of pregnancy. However, at a certain point, the labor onset signal is triggered, and uterine contractions are initiated. Inflammatory and endocrine pathways are generally acknowledged to mediate the “labor onset signal”—triggering event, causing uterine contractility and cervical dilation.^1^ When this event is triggered too early or too late, it may result in preterm birth or delayed birth.

The proposed mechanism responsible for parturition in the human uterus involves “functional” progesterone withdrawal and is mediated by decreased progesterone activity associated with alterations in the expression of progesterone receptors (PRs) and the ratios of PRA/PRB^2^ or PRC/PRB.^3^ Functional progesterone withdrawal involves crosstalk with inflammatory mediators in human parturition.^4^ Contraction-associated proteins (CAPs) in the uterus, including oxytocin receptor (OXTR), connexin 43 (Cx43), and cyclooxygenase 2 (Cox2), are the final effectors of PR and inflammatory mediators.^5^

Long noncoding RNAs (lncRNAs) are transcripts longer than 200 nucleotides that were previously considered to be “noise” in gene transcription.^6^ A large body of evidence has demonstrated that lncRNAs are engaged in signaling pathways in human physiological and pathological states. LncRNAs execute molecular functions as archetypes of decoys, signals, guides, and scaffolds.^7^ LncRNAs were reported to have critical roles in the development of pregnancy-associated diseases, such as preeclampsia,^8^^,^^9^ fetal growth restriction,^10^^,^^11^ and gestational diabetes mellitus.^12^^,^^13^ Luo et al. have reported the differential expression in myometrium during human parturition; however, the role of lncRNAs during human parturition has not been reported.^14^

In the current study, long non-coding RNA (lncRNA) growth arrest-specific 5 (GAS5) was identified to exhibit upregulation in the lower segment myometrium of individuals in labor compared to those not in labor, as determined by RNA sequencing.

N6-methyladenosine (m6A) is the most abundant internal modification of RNA in eukaryotic cells. The m6A modification affects multiple aspects of RNA metabolism, ranging from nuclear export, RNA processing, and RNA translation to decay.^15^ m6A is installed by m6A methyltransferases (METTL3/14, WTAP, etc., termed “writers”), removed by demethylases (FTO, ALKBH3/5, termed “erasers”), and recognized by m6A-binding proteins (YTHDC1/2, YTHDF1/2/3, IGF2BP1/2/3, etc., termed “readers”).^16^ Recent studies have reported that several lncRNAs, including MALAT1, MEG3, XIST, GAS5, and KCNK15-AS1, are subject to m6A modification.^17^ Here, we found that GAS5 RNA stability was increased by m6A modification and enhanced uterine muscle cell contraction, which triggers human parturition.

## Materials and Methods

### Samples

Myometrium was obtained from consenting women undergoing cesarean deliveries from patients during January 2022 to June 2022 in the Department of Gynecology and Obstetrics of the People’s Hospital of Jiangsu Province, China. Clinical characteristics are listed in Table 1. Samples were classified into 2 groups: not in labor (NIL, *n* = 13) and in labor (IL, *n* = 11). In labor was defined as women who had the presence of contractions of sufficient strength and frequency to effect progressive effacement and cervix dilation; not in labor was defined as women with no regular contractions, who underwent cesarean deliveries because of macrosomia and breech position. Uterine smooth muscle (2.0 × 0.5 × 0.5 cm^3^) was cut from the site of incision at the lower edges of the uterus during cesarean section. Samples were then washed 3 times with ice-cold sterile phosphate-buffered saline (PBS) and then divided into 2 pieces: one was stored in liquid nitrogen for RNA and protein extraction, and one was fixed in formaldehyde solution (4%) for immunohistochemical analysis. In some cases, similarly sized muscles were stored in cold PBS and used for in vitro cultures of myometrial cells. All experiments were approved by the Ethics Board of the First Affiliated Hospital of Nanjing Medical University.

**Table 1.** Clinical Characteristics of the Not in Labor (NIL) and In Labor (IL) Patients.

	IL	NIL	*t*	*P*-value
Age (years)	32 ± 2.9	32 ± 2.0	0.144	.887
BMI (Kg/m2)	26.4 ± 1.49	26.5 ± 1.31	−0.221	.827
Pregnant weeks	39.2 ± 0.43	39.1 ± 0.48	0.483	.633
Birth weight (g)	3476.7 ± 258.34	3426.7 ± 339.57	0.454	.653
Placental weight (g)	548.0 ± 27.4	542.7 ± 34.0	0.473	.640

Table 1 Clinical characteristics of the NIL and IL patients, including maternal age, gestational week, BMI, and weight of placenta and baby. There were no significant differences in these characteristics between the groups. All IL patients underwent normal labor prior to delivery.

### Human Myometrial Cell Culture

Uterine samples were obtained from women undergoing an elective term cesarean section delivery (≥39 weeks of gestation). These women had no signs of infection or any pregnancy complications, and they were defined as not in labor on the basis of a quiescent uterus, intact membranes, and a closed cervix. After delivery of the placenta, samples of myometrium (1 cm^3^) were excised from the upper incisional margin of the lower uterine segment and were immediately washed in ice-cold PBS. Myometrium was carefully minced into small pieces of about 1 mm^3^, subsequently incubated with gentle agitation for 2 h at 37°C, with collagenase IA (C9891, Sigma-Aldrich) and collagenase XI (C7657, Sigma-Aldrich) each at 0.5 mg/mL in Dulbecco’s modified Eagle medium (DMEM)/F-12 media (A4192001, Gibco) supplemented with BSA (sh30087.02, HyClone) at 1 mg/mL. The dispersed cells were separated from nondigested tissue by filtration through a cell strainer (70 µm, 431 751, Corning) and then collected by centrifugation of the filtrate at 3000 rpm for 5 min at room temperature. The cells were suspended in DMEM/F-12 media supplemented with 10% fetal bovine serum (10270-106, Gibco) and 1% penicillin/streptomycin/amphoterin B (15 240 062, Gibco) in a humidified atmosphere (5% CO2 in air). Culture medium was changed every 48-72 h. Experiments were performed using cells between passages 2 and 5.

### Cell Transfection

The siRNA interference sequences of GAS5, METTL3, IGF2BP1, and TPM4 are listed in Table S1. The full length of the human GAS5 (NR_002578.3), METTL3 (NM_019852.5), IGF2BP1 (NM_001160423.2), and TPM4 (NM_00329.3) were cloned into the pcDNA3.1(+) vector (Invitrogen # V80020). Lipofectamine 2000 (Invitrogen, Carlsbad, California, USA) was used for cell siRNA transfection, and the X-Tremegene HP DNA transfection reagent (Roche, Mannheim, Germany) was used for cell plasmid transfection. During transfection, 50%–70% of cells in the six-well plate should be transfected. Six hours after the siRNA transfection, the cells required a medium change, but not for the plasmid transfection. RNA was collected 24 h after cell transfection, and protein was collected 48 h after cell transfection.

### Total RNA Extraction and m6A-qRT-PCR

The total RNA was extracted from human primary myometrial cells using the Trizol reagent (Invitrogen, Carlsbad, California, USA). 100  µg of the total RNA was digested by DNase (Takara, Shiga, Japan) in a 150 µl reaction system at 37°C for 20  min. Then the total RNA was extracted again using the Trizol reagent, followed by RNA fragmentation using fragmentation reagents (Invitrogen, Carlsbad, California, USA) at 71°C for 5  min. A termination buffer was added immediately. The fragmented RNA was extracted using the Trizol reagent and dissolved in 200 µl of diethyl pyrocarbonate (DEPC) water. A volume of 160 µl of fragmented RNA was diluted with the MeRIP buffer (150 mm KCl, 25 mm Tris, 5 mM EDTA, 0.5% Triton X-100, 0.5 mm DTT, RNAase inhibitor (1:1000) (ABclonal, Wuhan, China) and protease inhibitor (1:100) (Invitrogen, Carlsbad, California, USA) and divided into 2 tubes that were incubated with the anti-m6A antibody (ABclonal, Wuhan, China) or the control IgG antibody with protein A/G conjugated magnetic beads (MCE, Monmouth Junction, NJ, USA) in 900 µL of the RNA binding protein immunoprecipitation (RIP) lysis buffer at 4°C for 4 h. In total, 20 µL of fragmented RNA was collected. The bound RNAs were immunoprecipitated with beads. The beads were washed with the RIP buffer and treated with 10 µL of 10% sodium dodecyl sulfate (SDS), 10 µL of proteinase K (Takara, Shiga, Japan), and 130 µL of the MeRIP buffer for 30 min at 55°C. Then the treated liquid was transferred to new tubes. In each tube, 1 mL of the Trizol reagent and chloroform were added in turn. After centrifugation, the upper water phase was collected. A 1/10 volume of 3 M sodium acetate and an equal volume of isopropyl alcohol and glycogen with a final concentration of 100 ug/mL were added. The samples were stored at −80°C overnight and then centrifuged at 12 000  ×  *g* at 4°C for 15 min. They were then washed with 75% ethanol. Finally, the precipitation was dissolved with DEPC water and analyzed using 2-step quantitative RT-PCR (Takara, Shiga, Japan).

### Quantitative Real-Time PCR

The plasmids of GAS5, METTL3, IGF2BP1, and TPM4 were extracted from *Escherichia coli* using the Endo free Plasmids Mini Kit II (50) Kit (OMEGA, Norcross, GA, USA). The total RNA was extracted from the myometrium cells or uterine samples using the Trizol reagent (Invitrogen, Carlsbad, California, USA), and the cDNA was reversed transcribed using the PrimeScript RT Reagent Kit (Takara, Shiga, Japan) according to the manufacturer’s protocol. Quantitative Real-Time PCR (qRT-PCR) was performed in triplicate in the Step One Plus TM real-time PCR Instrument (Applied Biosystems by Thermo Fisher Scientific, Singapore). GAPDH served as the internal reference gene. The primers are listed in Table S2.

### Western Blotting

Proteins were extracted from human uterine smooth muscle and human primary myometrial cells. Protein quantification was performed using BCA protein detection kit (23 229; Thermo Fisher Scientific). The protein was separated and transferred to nitrocellulose membranes (Millipore, Billerica, MA, USA). Followed by incubation with 5% milk and then incubated at 4°C with primary antibodies overnight. Detailed antibody information is listed in Table S3.

### Immunohistochemistry

Tissue samples from NIL and IL groups were fixed and then cut into 4 µm sections for the immunohistochemistry staining. The fixed paraffin-embedded sections were rehydrated in a graded series of decreasing alcohol concentrations. Sections were incubated in 3% hydrogen peroxide for 30 min to block endogenous peroxidase and 2% normal goat serum for 1 h at room temperature to reduce nonspecific binding. Then slides were incubated overnight at 4°C with primary antibodies. Detailed antibody information is listed in Table S3. Secondary antibody dilution on top for 2 h at room temperature. DAB chromogen solution was added so as to cover the entire tissue section and incubated for 10 min. The sections were rinsed in PBS, and then the slides were drained. The stained tissues were covered with a coverslip of an appropriate size and visualized under a microscope.

### Contraction Assay

Myometrium cells were harvested and resuspended in the desired medium at 2-5 × 106 cells/mL. Prepare the collagen lattice by minxing the cell suspension and cold collagen gel working solution according to the protocol (Cell Biolabs, San Diego, USA). A volume of 0.5 mL of the cell-collagen mixture per well was added in a 24-well plate, incubated for 1 h at 37°C. After collagen polymerization, 1.0 mL of culture medium was added atop each collagen gel lattice. Cultures were incubated for 2 days, and then the gel would be released with a sterile spatula. The gel size was measured by a ruler, and the gel area was quantified. Each experiment was analyzed in triplicate.

### RNA Binding Protein Immunoprecipitation

The myometrium cells were washed with ice-cold PBS and lysed in the RIP Lysis buffer (150 mM KCl, 25 mM Tris, 5 mM EDTA, 0.5% Triton X-100, 0.5 mM DTT, protease inhibitor (1:100), and RNAase inhibitor (1:1000) on ice for 30 min. The cell lysates were centrifuged at 12 000 × *g* at 4°C for 15 min. A total of 10% of the supernatant was collected, and the remaining supernatant was incubated with antibodies at 4°C for 4 h. Bound RNAs were immunoprecipitated with beads. The beads were washed with RIP buffer and treated with 10 µL of 10% SDS, 10 μL of proteinase K (Takara, Shiga, Japan), and 130 µL of the MeRIP buffer for 30 min at 55°C. RNA in the immunoprecipitation (IP) or input group was recovered with the Trizol reagent (Invitrogen, Carlsbad, California, USA) according to the manufacturer's instructions and analyzed by quantitative RT-PCR. The enrichment ratio was calculated as a ratio of its amount in the IP to that in the input. Detailed antibody information is listed in Table S3.

### RNA Stability Assays

Myometrium cells were seeded in six-well plates overnight, and METTL3, IGF2BP1 plasmids, and siRNA transfections were applied, respectively. Twenty-four hours later, the cells were treated with actinomycin D (5 μg/mL, HY-17559, MedChemExpress) at 0, 3, 6, and 9 h. The total RNA was then isolated by Trizol (Invitrogen, USA) and analyzed by qRT-PCR. The mRNA expression for each group at the indicated time was calculated and normalized by GAPDH. The mRNA half-lives time was estimated according to the linear regression analysis.

### Silver Staining and Mass Spectrometry

After 10% SDS-PAGE, the gel was maintained in a clean plastic 15-cm dish. Silver staining was performed by strictly following the manufacturer’s protocol (Pierce^®^ Silver Stain for Mass Spectrometry, 24 600, USA). When the band was visible, the developer working solution was immediately replaced with stop solution, and the gel bands were excised for further mass spectrometry (MS) analysis (Shanghai Applied Protein Technology Co., Ltd) on a Q Exactive mass spectrometer (Proxeon Biosystems, now Thermo Fisher Scientific).

### Fluorescence In Situ Hybridization and Subcellular Fractionation

Cy3-labeled GAS5 probes were obtained from RiboBio (Guangzhou, China). RNA Fluorescence in Situ Hybridization (FISH) was performed using a fluorescent in situ hybridization kit (Ribo Bio Tech). Fixed primary myometrium cells were permeabilized in PBS containing 0.5% Triton X-100 at 4°C for 5 min. Cells were washed with PBS 3 times for 5 min and prehybridized at 37°C for 30 min. Subsequently, a Cy3-labeled GAS5 probe was used in the hybridization solution at 37°C overnight in the dark. Cells were rinsed thrice in 4 × SSC with 0.1% Tween-20 for 5 min at 42°C in the next day, followed by washing once for 5 min at 42°C in 2 × SSC and then washed once for 5 min at 42°C. GAS5 probes were obtained from RiboBio (Guangzhou, China). RNA FISH was performed using a fluorescent in situ hybridization kit (RiboBio Tech). Fixed primary myometrium and analyzed with LAS AF Lite (Leica, Solms, Germany). Cytoplasmic and nuclear RNA were separated and purified using the PARIS Kit (Life Technologies, Carlsbad, CA, USA) according to the manufacturers’ instructions.

### Mice Model of in Labor and Not in Labor

All animal care and procedures were conducted in accordance with the standards of the Administrative Regulations on Laboratory Animals approved by the State Council of the People’s Republic of China. The animal experiments were approved by the Ethics Committee of Experimental Animal Welfare of the Nanjing Medical University. In this experiment, female CD-1 mice aged 8 weeks were purchased from the Vitonlievera experiment and maintained in the condition without pathogens. Female mice were mated overnight with males (1:1), and the day of vaginal plug detection was designated as the gestational day (GD) 0.5 of pregnancy. The average time of term delivery in our facility was GD 19–20 (GD, 19.5). Our criteria for labor were the delivery of at least 1 pup, and the pregnant mouse was sacrificed. Mice that did not deliver at term were sacrificed 24 h postterm (GD20.5). We recorded the maternal weight gain, litter size, and the fetal and placental weight. Maternal myometrium and placenta were collected for further analysis.

### Statistical Methods

Each experiment was performed with at least 3 biological replicates. Results are presented as the mean ± SD, comparisons were made using 2-tailed Student’ t-test, or a one-way analysis of variance and Bonferroni multiple comparisons test, *P*  <  .05 indicated statistical significance. Statistical analyses were compared using an unpaired 2-tailed Student’s t-test or a one-way analysis of variance and Bonferroni multiple comparisons test with SPSS software (version 25.0 for Windows, IBM Inc., Chicago, IL, USA), and the figures were generated using GraphPad Prism 8.0 and Adobe Illustrator.

## Results

### CAPs in the NIL and IL Myometrium

**Figure 1.**
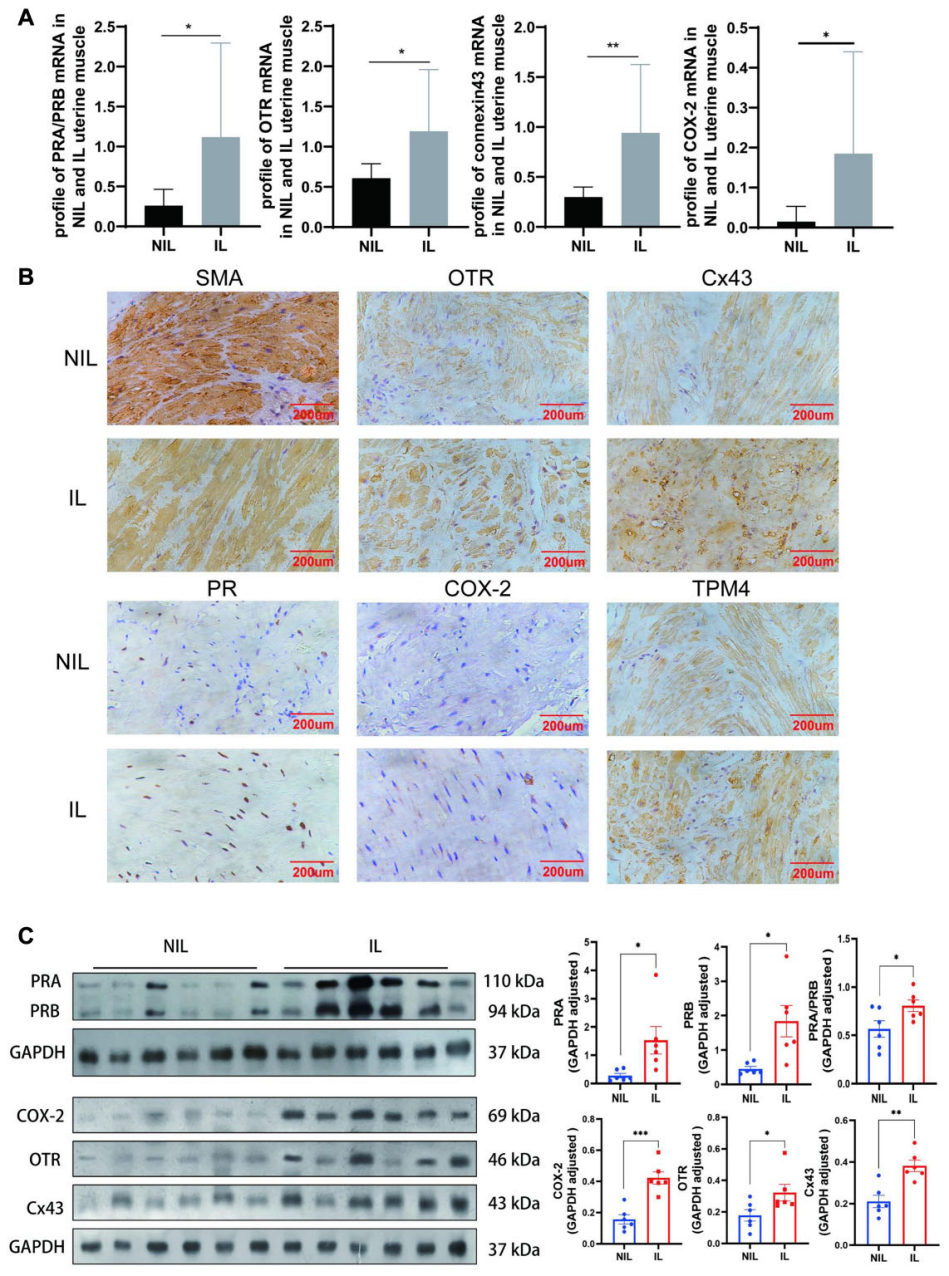
Contraction-associated proteins (CAPs) in not in labor (NIL) and in labor (IL) myometrium. (A) PRA/PRB mRNA ratio, CAPs mRNA (OTR, CX43, COX-2) were significantly higher detected in IL (*n* = 11) than in NIL (*n* = 13) group by quantitative real-time PCR (qRT-PCR). (B) Immunohistochemistry showed stronger staining of these CAPs and progesterone receptor (PR) in IL myometrium. (C) Western blotting also found increased expression of CAPs and PR in IL myometrium. (**P < .01, *P < .05). Bar, 200 µL.

Table 1 details the clinical features of patients in the NIL and IL groups. No significant differences in maternal age, gestational week, BMI, or weight of the placenta and baby were noted between the 2 groups. The PRA/PRB mRNA ratio and OXTR, Cx43, and Cox2 mRNA levels were significantly increased in the IL group (Figure 1A). PR, OXTR, Cx43, and Cox2 were also further assessed by immunohistochemistry staining, exhibiting stronger staining in IL uterine muscle (Figure 1B). Profiles of these proteins were also upregulated in IL uterine muscle compared with NIL muscles (Figure 1C).

### LncRNA Profiles in NIL and IL Myometrium

**Figure 2.**
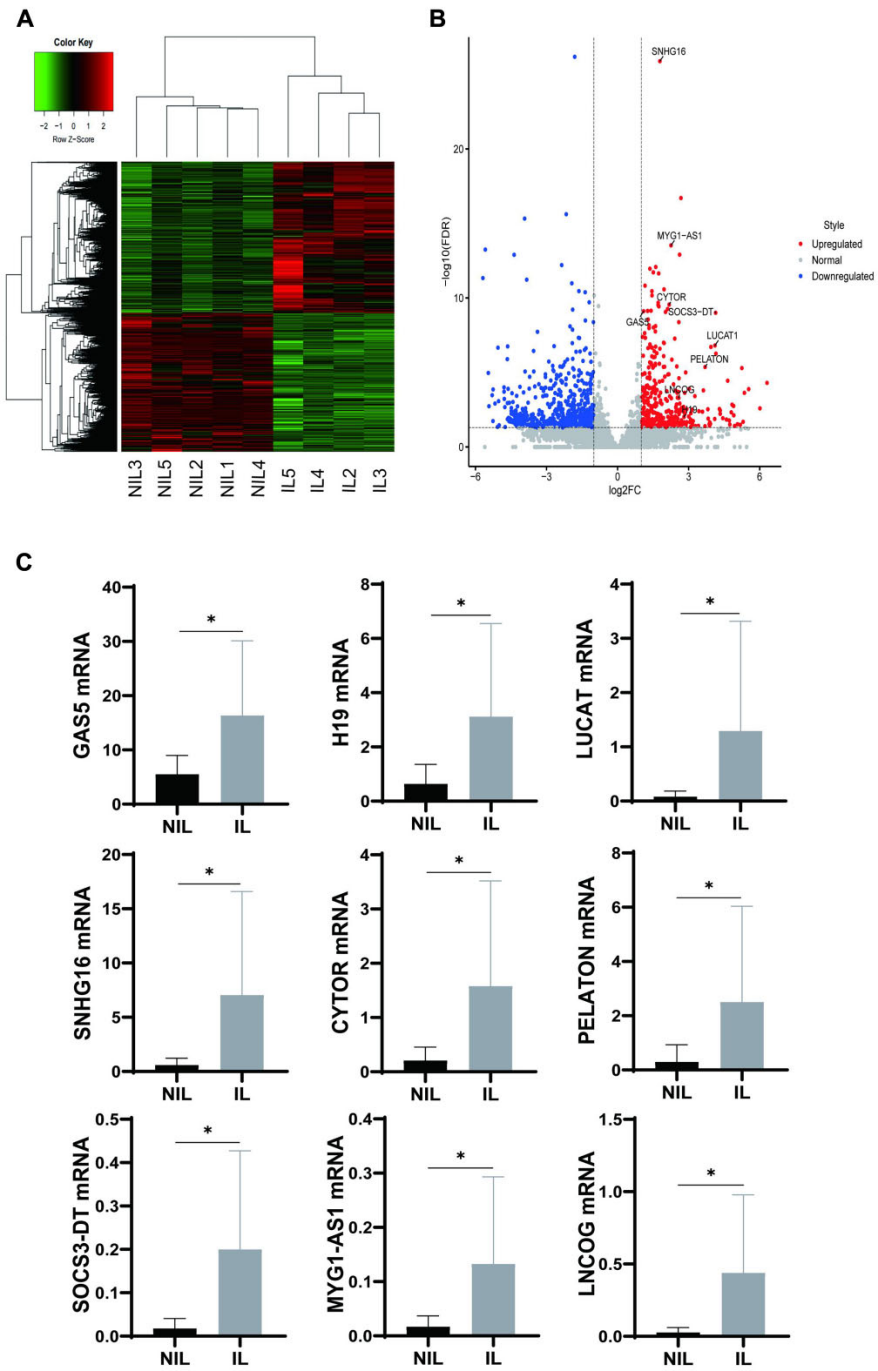
LncRNA profiles in human not in labor (NIL) and in labor (IL) myometrium. LncRNA sequencing was done in 2 groups: NIL (*n* = 5) and IL (*n* = 4). (A) The heat map in 2 groups. (B) The volcano plot of lncRNAs upregulated and downregulated in 2 groups: 205 lncRNAs were upregulated (*P *< .05) and 291 lncRNAs (*P *< .05) were downregulated. (C) 9 known upregulated lncRNAs were chosen: GAS5, PELATON, H19, SNHG16, LUCAT1, CYTOR, SOCS3-DT, MYG1-AS1, and LNCOG. Real-time PCR was done to validate the profile of these lncRNAs in NIL (*n* = 13) and IL (*n* = 11) myometrium. All the 9 lncRNAs were verified to be upregulated in IL myometrium. *P < .05.

RNA sequencing was performed in the NIL (*n* = 5) and IL (*n* = 4) myometrium. The heatmap is presented in Figure 2A. Volcano plot of genes upregulated and downregulated in IL vs. NIL group: 205 LncRNAs were upregulated (fold change > 2, *P *< .05), and 291 genes (fold change > 2, *P *< .05) were downregulated (Figure 2B). They are listed in Tables S4 and S5. Selection criteria: (1) upregulated LncRNA, (2) known LncRNA, (3) LncRNA expression level > 0 in each sample, (4) based on significance difference and fold change (top 20), exclude pseudogene and antisense strand, (5) related with muscle activity. Finally, 9 known upregulated lncRNAs were selected for further analysis: GAS5, PELATON, H19, SNHG16, LUCAT1, CYTOR, SOCS3-DT, MYG1-AS1, and LNCOG (Figure S2). Real-time PCR was performed to validate the profile of these lncRNAs in the NIL (*n* = 13) and IL (*n* = 11) myometrium. All 9 lncRNAs were verified to be upregulated in the IL myometrium (Figure 2C).

### GAS5 Promotes Contractions in Primary Myometrial Cells

**Figure 3.**
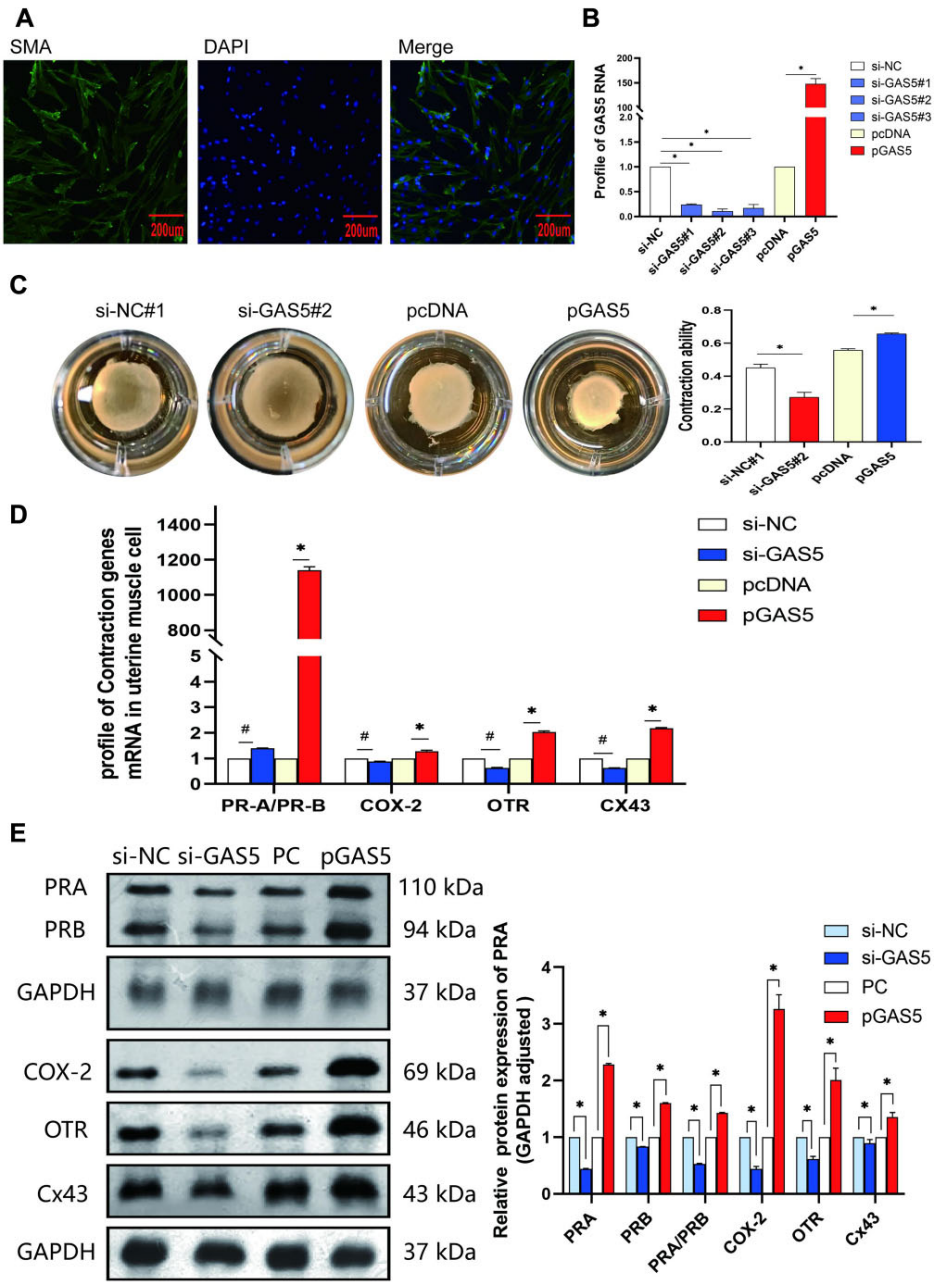
GAS5 promotes contractions in primary myometrium cells. (A) Human primary myometrium cells were extracted and stained by α-SMA antibody. 100% of cells were α-SMA positive. (B) Transfection efficiency in primary myometrium cells with specific GAS5 siRNAs and GAS5 overexpression plasmid. (C) 3D collagen gel matrix was tested in myometrium cells after GAS5 siRNAs and GAS5 plasmid transfected. (D) Progesterone receptors (PRs) and contraction-associated proteins (CAPs) mRNA were detected by quantitative real-time PCR (qRT-PCR). (E) PRs and CAPs proteins were analyzed by western blotting. The gray values were quantified with the analysis software. *P < .05. ^#^*P* < .05.

Human primary myometrial cells were extracted and stained with an α-SMA antibody. A total of 100% of the cells were α-SMA positive (Figure 3A). To clarify the effect of GAS5 on myometrial cell contractions, 3 specific GAS5 siRNAs and GAS5 overexpression plasmids were transfected into human primary myometrial cells (Figure 3B). GAS5 siRNA #2 showed the best efficiency, resulting in a 90% reduction. In contrast, GAS5 expression was significantly increased by 150-fold using the overexpression plasmid (Figure 3B). A 3D collagen gel matrix was applied to measure the gel area. The results showed that GAS5 overexpression strengthened the contraction of myometrial cells and that GAS5 siRNA inhibited contraction (Figure 3C). The PRA/PRB ratio and OXTR, Cx43, and Cox2 mRNA levels were significantly increased by GAS5 overexpression and decreased by GAS5 siRNA (Figure 3D). Western blotting results showed that PR, OXTR, Cx43, and Cox2 protein levels were also significantly elevated by GAS5 overexpression and decreased by GAS5 siRNA (Figure 3E).

### m6A RNA Modification Regulates GAS5 RNA Stability in Myometrial Cells

**Figure 4.**
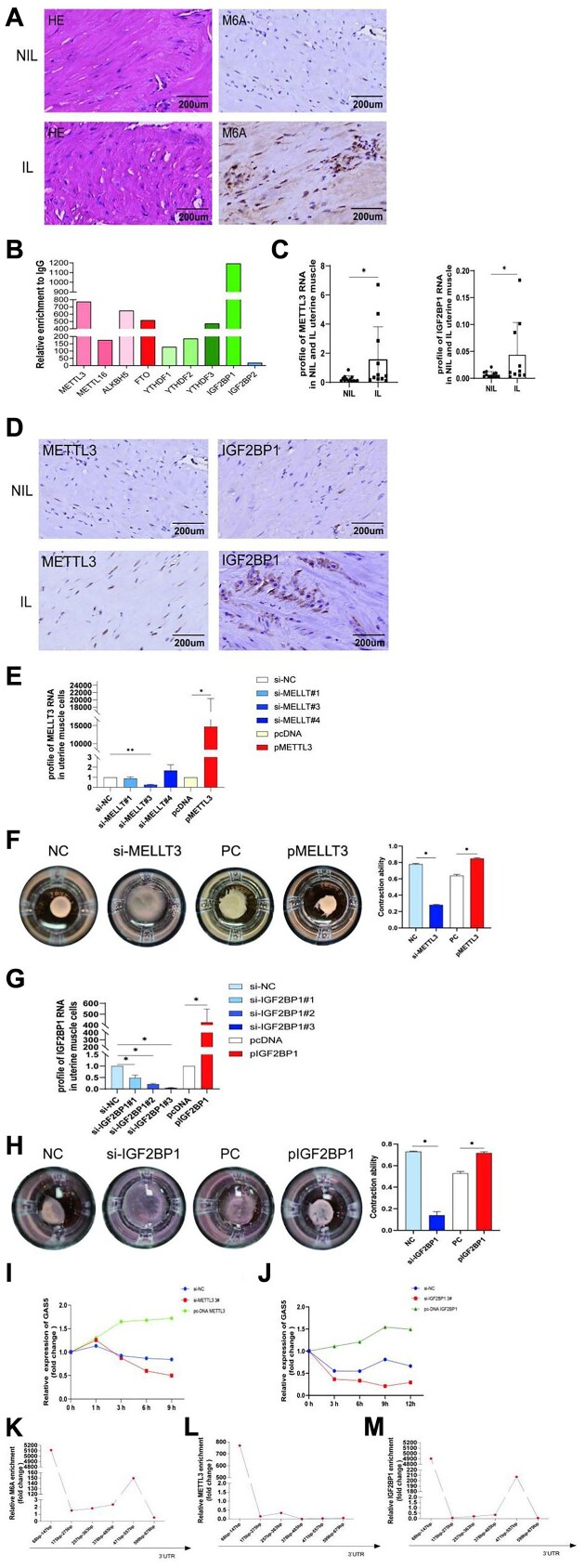
m6A RNA modification regulates GAS5 RNA stability and strengthens contraction ability of myometrium cells. (A) m6A immunohistochemistry staining in not in labor (NIL) and in labor (IL) myometrium. (B) RIP analyze was done to detect the underlying upstream regulating m6A enzymes, including METTL3, METTL16, AKKBH5, FTO, YTHDC1, YTHDF1/2/3, and IGF2BP1/2. (C) METTL3 and IGF2BP1 mRNA were detected higher expressed in IL myometrium than that in NIL myometrium by qRT-PCR. (D) METTL3 and IGF2BP1 immunohistochemistry staining in NIL and IL myometrium. (E) Transfection efficiency in primary myometrium cells with specific METTL3 siRNAs and its overexpression plasmid. (F) 3D Collagen gel matrix was tested in myometrium cells after METTL3 siRNA#3 and plasmid transfected. (G) Transfection efficiency in primary myometrium cells with specific IFG2BP1 siRNAs and its overexpression plasmid. (H) 3D collagen gel matrix applied after IFG2BP1 siRNA#3 and plasmid transfected. (I-J) Actinomycin test was done to find the effect of METTL3 and IGF2BP1 on GAS5 RNA stability. (K-M) MeRIP was done to find the specific conjugated sites of GAS5 RNA. IGF2BP1 enrichment was found to be at the 68-147 bp and 471-557 bp in the 5′ untranslated region (5′UTR) of GAS5. Mettl3 enrichment was found to be at the 68-147 bp in the 5′ untranslated region (5′UTR).

m6A immunohistochemistry staining was stronger in the IL myometrium (Figure 4A). RIP analysis was performed to detect the underlying upstream regulating m6A enzymes, including METTL3, METTL16, AKKBH5, YTHDC1, YTHDF1/2/3, and IGF2BP1/2. The writer METTL3 and reader IGF2BP1 were the top 2 significantly altered enzymes (Figure 4B). Furthermore, METTL3 and IGF2BP1 mRNA were noted to be more highly expressed in the IL myometrium than in the NIL myometrium (Figure 4C). And YTHDC2, YTHDC3, ALKBH3, ALKBH5, eIF3, FTO, IGF2BP2, IGF2BP3, METTL14, METTL16, WTAP, YTHDC1, and ZC3H13 mRNA were also detected in IL and NIL myometrium by qRT-PCR (Figure S1). Both enzymes showed stronger staining in the IL myometrium (Figure 4D). To clarify the effect of METTL3 and IGF2BP1 on myometrium cell contractions, specific siRNAs and plasmids were applied to transfect myometrium cells (Figure 4E, G), and contraction gels were measured. METTL3 and IGF2BP1 overexpression strengthened the contraction of myometrium cells, and their siRNAs inhibited cell contractions (Figure 4F, H). An actinomycin test was performed to determine the effect of METTL3 and IGF2BP1 on GAS5 RNA stability. GAS5 RNA stability was significantly increased by METTL3 and IGF2BP1 (Figure 4I, J). MeRIP was further performed to identify the specific sites of GAS5 RNA binding. IGF2BP1 enrichment was noted at the 68-147 bp and 471-557 bp sites of GAS5. Mettl3 enrichment was noted at the 68-147 bp site (Figure 4K, L, M).

### GAS5 Binding to Tropomyosin Alpha-4 (TPM4) in the Cytoplasm of Myometrium Cells

**Figure 5.**
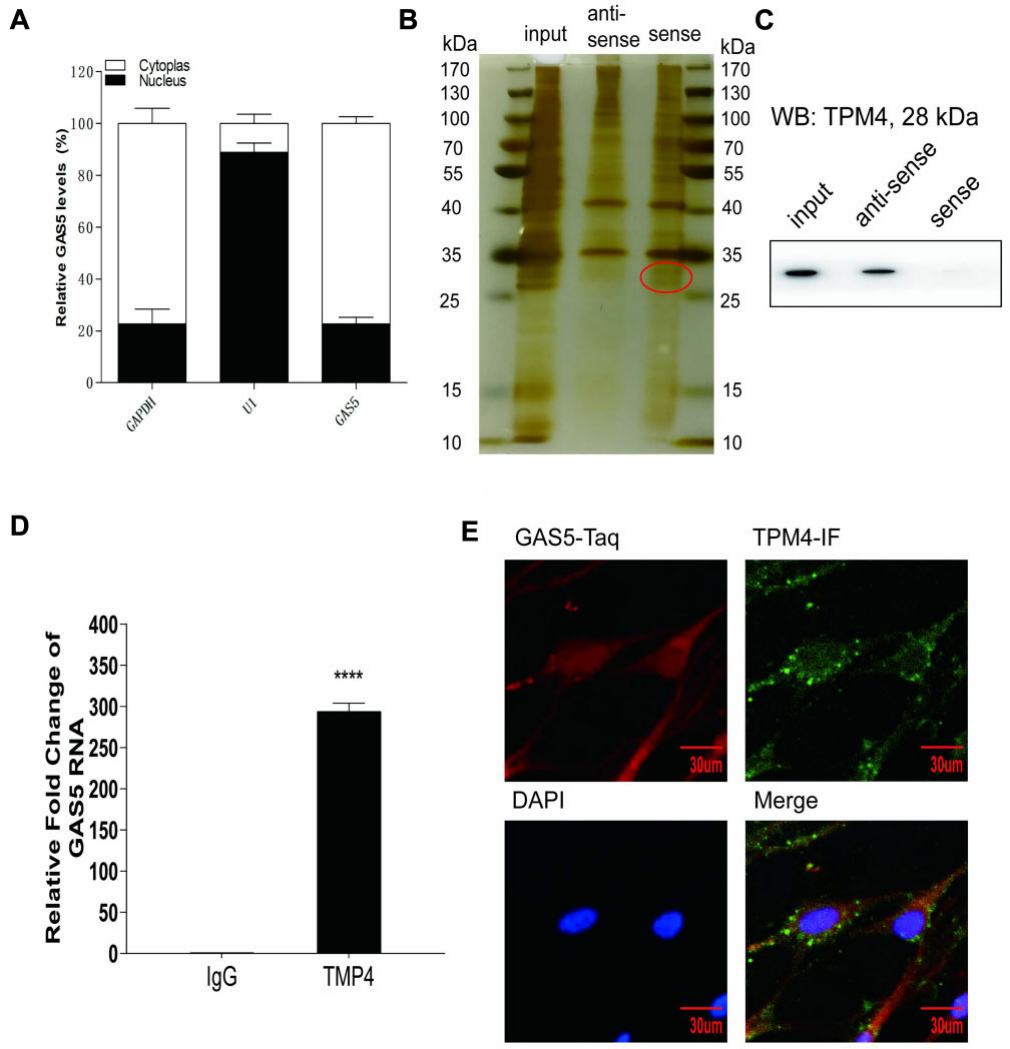
GAS5 binds TPM4 in cytoplasm of myometrium cells. (A) Distribution of GAS5 in myometrium cells. Approximately 90% of GAS5 was distributed in the cytoplasm. (B) RNA pull-down and silver staining were done to find GAS5 related binding proteins. (C) Western blotting was done to find TPM4 in the pulldown protein lysis. (D) RIP was conducted to find GAS5 integrating TPM4. (E) Immunofluorescence staining for TPM4 (green) and FISH staining for GAS5 (red) and DAPI staining for nucleus (blue) were conducted in the same myometrium cell fixed slide, indicating the binding of GAS5 and TPM4 in cytoplasm of myometrium cells. ^****^P < .0001.

To investigate the downstream underlying mechanism of GAS5, we analyzed the cytoplasmic and nucleus distributions of GAS5 in myometrial cells by qPCR. The results showed that approximately 90% of GAS5 was distributed in the cytoplasm (Figure 5A). RNA pulldown and silver staining were performed to identify GAS5-related binding proteins. We found missing bands between 25 and 35 kDa in the GAS5 antisense lane (Figure 5B). The antisense and sense gels were further analyzed by protein MS. TPM4 was not identified in the antisense gel; its molecular weight was 28 kD (Figure 5C). RIP was conducted to find GAS5 binding to TPM4 (Figure 5D). Furthermore, immunofluorescence staining for TPM4 (green), FISH staining for GAS5 (red), and DAPI staining for the nucleus (blue) were conducted in the same myometrium slide sample (Figure 5E). We observed orange light in the merged slides (Figure 5E), demonstrating the coexpression of GAS5 and TPM4 in the cytoplasm and indicating the coexpression of GAS5 and TPM4 in the cytoplasm of myometrial cells.

### TPM4 Promotes Contractions in Primary Myometrium Cells

**Figure 6.**
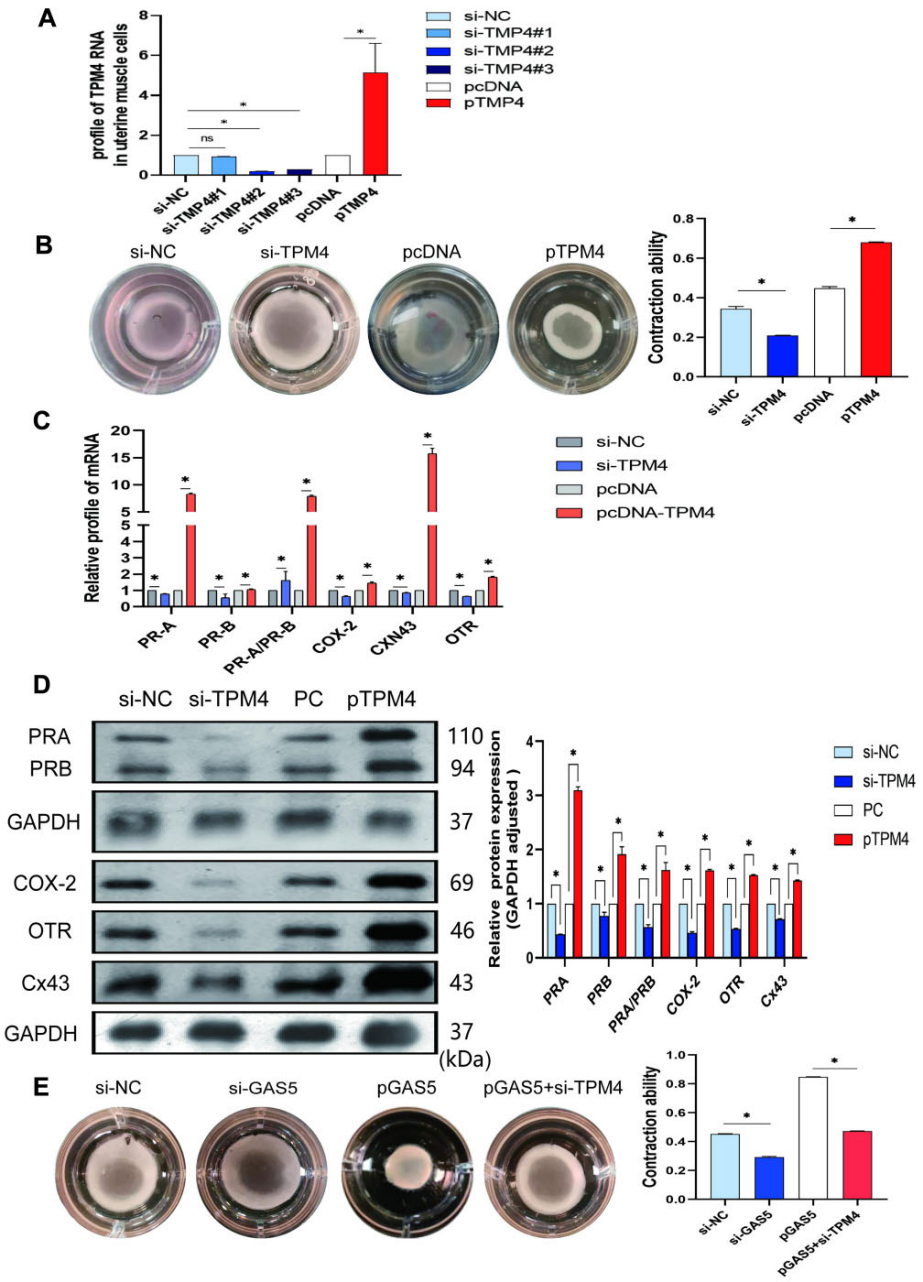
TPM4 promotes contractions in primary myometrium cells. (A) Transfection efficiency in primary myometrium cells with specific TPM4 siRNAs and its overexpression plasmid. (B) 3D collagen gel matrix applied after TPM4 siRNA#2 and plasmid transfected. (C) Progesterone receptors (PRs) and contraction-associated proteins (CAPs) mRNA were detected by quantitative real-time PCR (qRT-PCR). (D) PRs and CAPs proteins were analyzed by western blotting. The gray values were quantified with the analysis software. (E) Rescue experiment was done by using GAS5 plasmid and TPM4 siRNA, and the contraction ability was significantly inhibited (Figure 6E). *P < .05, ^#^*P* < .05.

To clarify the effect of TPM4 on myometrial cell contractions, 3 specific siRNAs and an overexpression plasmid were transfected into human primary myometrial cells. TPM4 siRNA #2 showed the best efficiency, yielding an 80% reduction in TPM4 expression, whereas the TPM4 expression was significantly increased by 5-fold using the overexpression plasmid (Figure 6A). The 3D collagen gel matrix results showed that TPM4 overexpression strengthened the contraction of myometrium cells, and TPM4 siRNA inhibited the contractions (Figure 6B). The PRA/PRB ratio and CAP mRNA levels were significantly increased upon TPM4 overexpression, whereas CAP mRNA levels were decreased by TPM4 siRNA (Figure 6C). Western blotting results showed that PR and CAP protein levels were also significantly elevated by TPM4 overexpression and decreased by TPM4 siRNA (Figure 6D). The rescue experiment demonstrated that si-TPM4 can attenuate the ability of GAS55 to promote uterine smooth muscle cell contraction (Figure 6E).

### CAPs and m6A in the Myometrium of NIL and IL Mice

**Figure 7.**
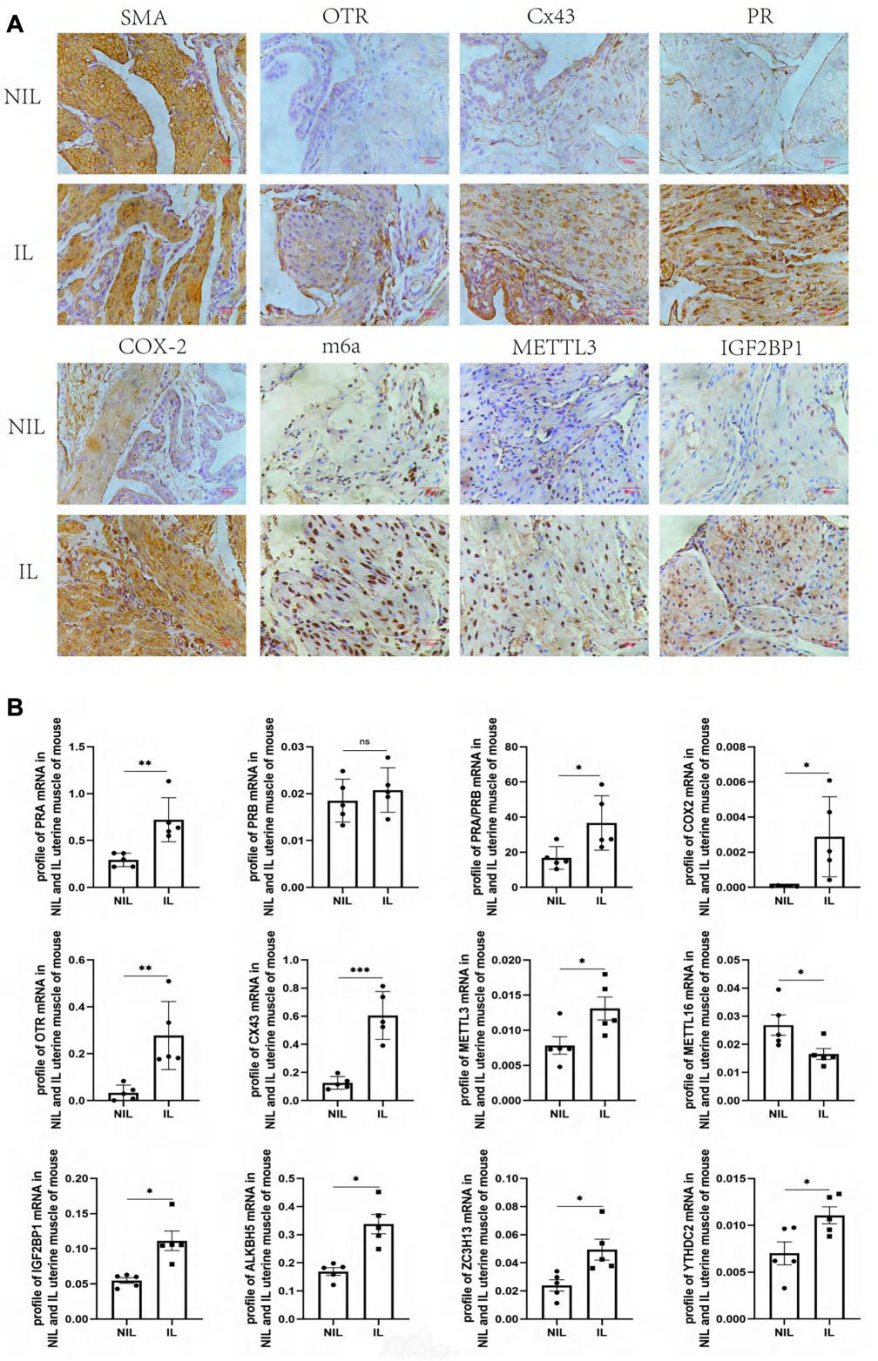
Contraction-associated proteins (CAPs) and m6A in not in labor (NIL) and in labor (IL) mice myometrium. (A) CAPs, PR, and m6A immunochemistry staining in IL mice myometrium. (B) CAPs, PR, METTL3, METTL16, and IGF2BP1, ALKBH5, ZC3H13, and YTHDC2 mRNA were detected by quantitative real-time PCR (qRT-PCR) in NIL and IL mice myometrium.

There are no differences in litter size, fetal weight, placental weight, weight of maternal mice in NIL and IL mice (Figure S1A). m6A, CAPs, and PR were detected by immunochemistry staining, demonstrating stronger staining in IL mouse uteri compared with NIL controls (Figure 7A). CAPs and PR mRNA and m6A enzymes (METTL3, METTL16, IGF2BP1, ALKBH5, ZC3H13, and YTHDC2) mRNA were also significantly increased in the IL group compared with the NIL controls (Figure 7B). Other mRNA enzymes, including ALKBH3, METTL14, THDC1, YTHDF1, YTHDF2, YTHDF3, VIRMA, FTO, WTAP, IGF2BP2, and IGF2BP3, were found to have no difference in both groups (Figure S1B).

## Discussion

Labor signaling initiation is a complex process in mammals involving myometrial activation, cervical ripening, and dilation. The transition of the myometrium from quiescence to the highly contractile state is thought to be controlled at the transcriptional and posttranscriptional levels, including mRNA and ncRNA regulation.^18^ A variety of differentially expressed coding genes between the myometrium not in labor and in labor myometrium have been reported in previous studies.^19^^,^^20^ LncRNAs have novel regulatory roles in the posttranscriptional modification of coding genes and may serve as potential prognostic and therapeutic markers in human diseases.^21^^,^^22^ LncRNA sequencing was performed by Luo et al., revealing 69 upregulated lncRNAs and 43 downregulated lncRNAs in the IL myometrium, such as SNHG3/8/15, PGM5-AS1, and loc107985064.^14^ However, the mechanistic role of lncRNAs in the regulation of myometrium contractions remains unclear.

In the present study, lncRNA sequencing was also performed, and 9 identified lncRNAs upregulated in labor myometrium were chosen. GAS5 exhibited abundant levels in the labor myometrium. High expression of Caps is evidence of uterine contraction, so in the full text, we use it as a marker to detect uterine contraction.^5^ When GAS5 was upregulated or downregulated, the contractions of myometrium cells and CAPs were regulated accordingly, confirming the role of GAS5 in uterine contractions. GAS5 accumulates inside cells in response to cellular growth arrest and functions as a potent repressor of the glucocorticoid receptor through its RNA “glucocorticoid response element (GRE).”^23^ The progesterone receptor also has the GRE element, so we intended to identify a regulatory relationship between GAS5 and PR. However, PR isoforms were not observed in the MS results, and PR protein bands were not detected in the RIP results. Instead, TPM4 was shown to bind with GAS5 in myometrial cells by MS and RIP. Myometrium contraction involves the interaction of the protein filaments myosin and actin. In muscles, projections on the myosin filaments, which are also called myosin heads or cross-bridges, interact with the nearby actin filaments; myosin filaments move the actin filaments using a cyclic rowing action to produce muscular movements.^24^ GAS5 regulates complex intracellular signaling pathways primarily through 3 modes of action, including signal, decoy, and guide; all of these processes are related.^25^ In this study, we found binding of GAS5 and TPM4 in the cytoplasm of myometrial cells, which might be a new role of the GAS5 scaffold. This is the first report of the mechanism of lncRNA GAS5 in human parturition. We also noted that CAP genes also exhibit changes concomitant with those of TPM4; however, this does not imply that CAP genes are downstream of TPM4. And this needs to be further investigated. N6-methyladenosine (m6A) RNA methylation is the most prevalent posttranscriptional modification mechanism in humans. m6A modifications are noted in genes related to human^26^ and porcine^27^ placental development. Taniguchi et al. suggested that m6A both at the 5′-UTR and in the vicinity of the stop codon in placental mRNA played important roles in fetal growth and preeclampsia.^26^ In our research, we assessed the m6A profile in human IL and NIL myometrium, revealing stronger staining in IL myometrium. The “writer” METTL3 and the “reader” IGF2BP1 were confirmed to bind with GAS5 by RIP and MeRIP-qPCR, respectively. GAS5 was reported to be negatively regulated by the m6A reader YTHDF3 and involved in the progression of colorectal cancer.^28^ In addition to being a “writer” and “reader,” the “eraser” FTO was also reported to reduce the m6A modification of GAS5 and promote the EMT process and inflammatory response in the process of renal interstitial fibrosis.^29^ However, in the highly contractile state of labor onset, METTL3 and IGF2BP1 exhibit different GAS5 modifications compared with YTHDF3 and FTO. These factors exhibited a crucial role in maintaining the stability of GAS5. As a crucial methyltransferase in m6A modification, METTL3 regulates many lncRNAs, such as MALAT1,^30^ XIST,^31^ and SNHG1.^32^ Studies have also reported that IGF2BP1 plays essential roles in the regulation of many lncRNAs, such as LINC00483^33^ and KB-1980E6.3.^34^ Furthermore, in this study, we also detected the binding site of GAS5, 68-147 bp in the 5′-UTR of METTL3 and 68-147 bp and 471-557 bp in the 5'-UTR of IGF2BP1.

To study whether m6A modifications exist in mouse parturition, we generated a labor onset mouse model. Myometrium was collected from mice in labor and those not in labor, and we compared the profiles of m6A, METTL3, IGF2BP1, and CAPs in these tissues. The same trend noted in the human myometrium was observed in mouse myometrium. However, GAS5 exhibits no homology between mice and humans, and the mechanism of GAS5 could not be verified in mice. Despite this limitation, we provide important clues for the m6A modification in the mechanism of parturition as well as crucial topics for future research, especially at the mouse model level.

In conclusion, GAS5 was first found to exhibit increased expression in the myometrium of humans in labor based on lncRNA sequencing and bioinformatics methods. Our study revealed a novel m6A modification of lncRNA GAS5, highlighting the need for further study of lncRNAs in human parturition.

## Supplementary Material

zqaf009_Supplemental_Files

## Data Availability

The datasets used and analyzed during the current study are included in this paper or are available from the corresponding authors upon reasonable request. To review GEO accession GSE236038: Go to https://www.ncbi.nlm.nih.gov/geo/query/acc.cgi?acc=GSE236038. Enter the token gdkvyaeidlwftwn into the box.
