# Supplementary material for: LncRNA GAS5 Regulates Myometrial Cell Contractions in an m6A-Dependent Manner
Source: Function (Oxf). 2025 Mar 7;6(2):zqaf009. doi: 10.1093/function/zqaf009 (PMC11931615; doi:10.1093/function/zqaf009)
Supplement: zqaf009_Supplemental_Files [file zqaf009_supplemental_files.zip › Table S2.docx]

**Table S2** The primers used in this study

| Human Gene | Sequence (5'-3') |
| --- | --- |
| GAS5 | Forward primer TGATTGCCTTGGTGAGTCAA  Reverse primer TCAAGCCGACTCTCCATACC |
| TMP4 | Forward primer CACGTATCGGCTTTGTTTGA  Reverse primer GGGTGGGTAAGGGATTCAGA |
| PRA | Forward primer TGCAAGGCTGATGGAAGACT  Reverse primer CCCACCACTGGATTTGACTC |
| PRB | Forward primer ACCCTTACGGATTCCTGGAG  Reverse primer TAGGAGGGTTGCTTCCTTCA |
| OXTR | Forward primer ACAAGAACGAGTGTCGGTGA  Reverse primer AGTGGCATTCCTGGGTCATA |
| Cx43 | Forward primer TGTCCCTGGCCTTGAATATC  Reverse primer GTGAGGAGCAGCCATTGAA |
| Cox2 | Forward primer AGGAGCACGTCCAGGAACT  Reverse primer CCGGGTACAATCGCACTTAT |
| GAPDH | Forward primerTGGTATGAGAGCTGGGGAATG  Reverse primerCCTCCCCACCTTGAAAGGAA |
| METTL3 | Forward primer TTGTCTCCAACCTTCCGTAGT  Reverse primer CCAGATCAGAGAGGTGGTGTAG |
| METTL16 | Forward primer AGGGAGTAAACTCACGAAATCCT  Reverse primer AACCCCTTGTATGCGAAGCTC |
| ALKBH5 | Forward primer CGGCGAAGGCTACACTTACG  Reverse primer CCACCAGCTTTTGGATCACCA |
| YTHDC1 | Forward primer AACTGGTTTCTAAGCCACTGAGC  Reverse primer GGAGGCACTACTTGATAGACGA |
| YTHDF2 | Forward primer AGCCCCACTTCCTACCAGATG  Reverse primer TGAGAACTGTTATTTCCCCATGC |
| YTHDF3 | Forward primer TCAGAGTAACAGCTATCCACCA  Reverse primer GGTTGTCAGATATGGCATAGGCT |
| IGF2BP1 | Forward primer GCGGCCAGTTCTTGGTCAA  Reverse primer TTGGGCACCGAATGTTCAATC |
| IGF2BP2 | Forward primer AGTGGAATTGCATGGGAAAATCA  Reverse primer CAACGGCGGTTTCTGTGTC |
| FTO | Forward primer ACTTGGCTCCCTTATCTGACC  Reverse primer TGTGCAGTGTGAGAAAGGCTT |
| LUCAT1 | Forward primer TGTGTCCAAATGCTGTCCTC  Reverse primer GGGTTGCCTCTGTTTATCCA |
| SOCS3-DT | Forward primer AATGTTACGTGGCTGCATGA  Reverse primer AGGTGTCTCCACCCACATTT |
| H19 | Forward primer ATGGTGCTACCCAGCTCAAG  Reverse primer CTGTTCCGATGGTGTCTTTG |
| LNCOG | Forward primer CCTCACAGGTTGGCTGTCTT  Reverse primer ATTCAGGCCCTTCTCATCCT |
| CYTOR | Forward primer ACTCATGCCCAAAGTTACGG  Reverse primer TTATTCGAGGGATGCAGACG |
| MYG1-AS1 | Forward primer TCATCACCAGTCCCTTGCTT  Reverse primer ACAGCCGTCATGGAAATGAT |
| SNHG16 | Forward primer GATGCCGTCTTGTGTTTCCT  Reverse primer TGATTGCCTTGGTGAGTCAA |
| PLK1 | Forward primer ACCAGCACGTCGTAGGATTC  Reverse primer ATAACTCGGTTTCGGTGCAG |
| PELATON | Forward primer GCTGACTGCCTGAATGAACA  Reverse primer CAGTTTCCAAGACGCCAACT |
|  |  |
| Cx43 | Forward primer CCTTTGACTTCAGCCTCCAA  Reverse primer GCGAAAGGCAGACTGTTCAT |
| Cox2 | Forward primer GGTCATGGTGGAGAGGTGTA  Reverse primer GTCGCACACTCTGTTGTGCT |
| h-β-actin | Forward primer CAGACTGCGACACATTCCAT  Reverse primer CTCCTTCTTGCTCAGGGTGT |
| METTL14 | Forward primer CTGAGAGTGCGGATAGCATTG  Reverse primer GAGCAGATGTATCATAGGAAGCC |
| WTAP | Forward primer TAGACCCAGCGATCAACTTGT  Reverse primer CCTGTTTGGCTATCAGGCGTA |
| VIRMA | Forward primer ATGTCATGGAAACTGCACCTC  Reverse primer GAGTGCTGAAAACCAAACCCA |
| ZC3H13 | Forward primer ATCCCGAAGACCTAGCGTATT  Reverse primer TGAAGGGCCATGTATGAACCT |
| ALKBH3 | Forward primer GAGCCAGTCTGCTACTCAGC  Reverse primer AACACAAATTGTCGGTCACATTG |
| YTHDC2 | Forward primer ACCGACTAAGTCAATCTCTTGGT  Reverse primer AGGCTCCTAACAGCATGTTTTG |
| IGF2BP3 | Forward primer CCTGGTGAAGACGGGCTAC  Reverse primer TCAACTTCCATCGGTTTCCCA |
| Eif3a | Forward primer AGCCTGCCCTGGATGTTCT  Reverse primer GCCAAGTGGCTCTTACGAAGAT |
