# Supplementary material for: LncRNA GAS5 Regulates Myometrial Cell Contractions in an m6A-Dependent Manner
Source: Function (Oxf). 2025 Mar 7;6(2):zqaf009. doi: 10.1093/function/zqaf009 (PMC11931615; doi:10.1093/function/zqaf009)
Supplement: zqaf009_Supplemental_Files [file zqaf009_supplemental_files.zip › table S3.docx]

**Table S3**

**Western blotting antibodies**

anti-PR antibody (1:1000, AB32085, Abcam,Cambridge, MA, USA), anti-OXTR antibody (1:1000,23045-1-AP, Proteintech, Wuhan, China), anti-Cx43antibody (1:1000, A11752, ABclonal technology, Wuhan, China ), anti-COX-2 antibody (1:1000, AB179800, Abcam,Cambridge, MA, USA) and GAPDH (1:1000, Beijing Zhongshan Golden Bridge Biotechnology Co. Ltd., Beijing, China). The second antibody was goat anti-rabbit(1:5000, AS014, Abclonal technology, Wuhan, China), and the membrane was incubated at room temperature for 1h. The protein expression was observed using a chemiluminescence gel imaging system (Tanon 5200,Shanghai, China).

**RIP antibodies**

anti-METTL3(Ab195352, Abcam, USA),

anti-METTL16 (Ab252420, Abcam, USA),

anti-ALKBH5 (ABE547, MERCK, USA),

anti-FTO (ABE552, MERCK, USA),

anti-YTHDC1 (Ab264375, Abcam, USA),

anti-YTHDF1 (Ab220162, Abcam, USA),

anti-YTHDF2 (Ab220163, Abcam, USA),

anti-YTHDF3 (Ab220161, Abcam, USA),

anti-IGF2BP1 (Ab184305, Abcam, USA),

anti-IGF2BP2 (Ab128175, Abcam, USA),

and anti-IGF2BP3 (Ab177477, Abcam, USA)

**IHC antibodies**

m6A antibody (1:200, AB151230, Abcam, Cambridge, MA,USA), METTL3 antibody (1:500, AB195352, Abcam, Cambridge, MA, USA), and IGF2BP1 antibody (1:4000, AB229700, Abcam, Cambridge, MA, USA),anti-PR antibody (1:1000, AB32085, Abcam,Cambridge, MA, USA), anti-OXTR antibody (1:1000,23045-1-AP, Proteintech, Wuhan, China), anti-Cx43antibody (1:1000, A11752, ABclonal technology, Wuhan, China ), anti-COX-2 antibody (1:1000, AB179800, Abcam,Cambridge, MA, USA) .
