# Supplementary material for: LncRNA GAS5 Regulates Myometrial Cell Contractions in an m6A-Dependent Manner
Source: Function (Oxf). 2025 Mar 7;6(2):zqaf009. doi: 10.1093/function/zqaf009 (PMC11931615; doi:10.1093/function/zqaf009)
Supplement: zqaf009_Supplemental_Files [file zqaf009_supplemental_files.zip › Table S5.docx]

| upregulated | downregulated |
| --- | --- |
| NFE4,CD300H,C15orf54,CT69,IRAG1-AS1,LINC01537,C3orf86,LINC01050,PRELID2P1,LINC01127,MIR3945HG,SOCS3-DT,LUCAT1,PDE2A-AS2,MIR217HG,PELATON,LINP1,MIX23P3,LINC00311,ZNF503-AS1,UBE2SP1,LINC00460,RPL21P119,LINC02207,RPL13AP20,NAMPTP1,NME2P1,H19,HNRNPA1P4,TREML3P,LINC00937,MAP3K4-AS1,PRELID1P5,CIRBP-AS1,RPS26P6,FAM157A,IL10RB-DT,ARHGAP29-AS1,LNCOG,C9orf92,LINC01506,VPS9D1-AS1,OR52K3P,EIF4A2P1,LINC01943,NACA3P,PRKAR1B-AS1,HMGN2P46,RPL24P2,RPS3AP5,PPIAP31,RPS7P10,ARPC3P1,MYG1-AS1,RPS24P19,NPM1P6,RPS23P8,NAV2-AS2,CYTOR,HAGLROS,PSME2P2,WBP1LP2,FENDRR,FTH1P2,LINC01303,RPL37P23,RPL4P6,WDR5-DT,LINC00482,HK2-DT,DIRC1,CARD16,SNHG8,RPSAP19,RPL21P16,RPL21P28,FTH1P11,FTH1P10,LINC01465,CEP250-AS1,LINC00545,FAM207BP,RPL23AP42,C3orf36,SNHG16,CCR5AS,RPL24P8,PPP1R14BP3,PPP1R14B-AS1,HOTTIP,SNHG3,SH3RF3-AS1,RPL18AP3,LINC02785,GRASLND,ZFAS1,RPL13AP7,PCED1B-AS1,RPL32P18,LINC01503,LINC02432,RPLP0P6,RPS28P7,HSPD1P1,PCAT19,IFITM3P2,HNRNPA1P35,RPL21P75,RPS27AP16,NDUFA4P1,FLJ31356,RPL13P12,RPS15P4,NAV2-AS6,PRELID1P6,FTH1P8,DLGAP1-AS2,LSP1P4,EML2-AS1,SNHG15,PVT1,LINC01426,PSME2P1,FTH1P7,RPL7P23,NUP153-AS1,COSMOC,ZNF295-AS1,ST3GAL1-DT,LNCSRLR,C19orf48,RPL9P7,RPL6P27,EEF1B2P3,RPS3AP6,RPL13AP25,SNRPA1P1,SNRPGP2,RPS2P46,TPI1P1,SNHG1,LINC00623,RPSAP58,LINC00877,SNRPGP15,RPS7P1,RPL7AP30,RPL7P1,FTLP3,ARPC3P3,RPL3P4,EIF4A1P10,RPS20P14,ZNF232-AS1,RPS3AP26,RPL13AP5,RPL12P12,GORAB-AS1,RPL34P18,RPL37AP1,UBAC2-AS1,OSMR-AS1,RPL4P4,RPL35P1,RPL10AP6,PAPOLA-DT,RPS2P5,RPS7P11,RPL29P11,SNHG17,NCF1C,RPS4XP11,LINC01013,LINC01615,RPL22P1,RARA-AS1,RPL5P34,SNHG29,RPL17P50,C22orf34,RPL7P9,RPS13P2,ELOCP2,WASH9P,SNHG6,TPT1P9,SIGLEC16,GPX1P1,PTP4A2P1,GAS5,SNHG7,PPIAP29,KDM7A-DT,MIR155HG,AGAP2-AS1,RPL26P19,RANP1,RPL14P1,CARD8-AS1,DANCR,PRKAG2-AS1,CDC26P1,MED14OS,SNHG19,PA2G4P4 | PAXIP1-DT,RPL32P3,LINC00899,GTF2IP1,AHSA2P,PPP3CB-AS1,HULC,POM121L9P,ST13P6,LINC00856,HERC2P9,HCG11,LINC01197,KLF3-AS1,ZBED3-AS1,HERC2P2,FBXL19-AS1,HERPUD2-AS1,ERVK13-1,THAP7-AS1,MYOSLID,BACE1-AS,MTMR9LP,LINC00342,SGMS1-AS1,STX18-AS1,EMX2OS,TAGAP-AS1,USP32P3,CASC2,ITGB1P1,SEPTIN7P13,XIST,ANO7L1,TMEM167B-DT,BTN2A3P,NBPF8,SNHG14,FAM225B,LINC01534,BRD7P2,LINC01579,CASTOR3,RNF213-AS1,GUSBP11,STAG3L3,LINC00630,DUBR,TAPT1-AS1,TMEM220-AS1,SEPTIN7-DT,LINC00641,ARHGEF34P,XPC-AS1,PPM1F-AS1,LINC02606,MRPS30-DT,PCOLCE-AS1,USP27X-DT,A2M-AS1,TMEM30A-DT,LINC02767,PRECSIT,LINC00598,KCNQ1OT1,ZNF37BP,ZMIZ1-AS1,C22orf46,FAM198B-AS1,LINC00933,EIF4BP7,PLBD1-AS1,RUSC1-AS1,LINC00910,SRRM2-AS1,ATXN1-AS1,GTF2IP4,PDPR2P,LINC00989,LINC02268,LINC01480,CROCCP3,KMT5AP2,SSPOP,EIF2AK3-DT,TPTEP2,LINC02175,STAG3L1,CLEC4GP1,USP46-DT,MXRA7P1,ADIRF-AS1,BDNF-AS,LINC01376,NPEPPSP1,TMEM9B-AS1,LINC02349,LINC00894,LINC02202,NR2F2-AS1,CACTIN-AS1,ZNF137P,FAM66D,FBXO30-DT,SETBP1-DT,DUSP8P5,FAR2P2,MANEA-DT,STAG3L2,PAXIP1-AS2,TMEM254-AS1,LINC01273,CYP2T1P,LINC01719,MIR1-1HG-AS1,ZNRD1ASP,KMT5AP1,SUCLG2-AS1,SRGAP2D,LINC01088,AHCTF1P1,POT1-AS1,LINC01522,ZNRF2P1,HYDIN2,ZNF252P-AS1,PPP1R26-AS1,LINC00472,GAS6-AS1,LINC02884,HAND2-AS1,LINC01836,LINC02185,LINC00865,LINC01238,CFL1P1,ADH5P4,CT75,RTCA-AS1,NDUFA5P11,ADAMTS9-AS2,MIOS-DT,WEE2-AS1,POLR2J4,LINC00654,ST13P5,GABPAP,FLJ46284,LINC00996,UBE2Q2P1,LINC00886,AIDAP1,SUGT1P1,RAMP2-AS1,LINC01135,GOLGA6L5P,LINC02256,TLK2P1,NFYC-AS1,MEIS3P1,ANKRD36BP2,DNM1P35,SPTLC1P1,AOC4P,CYP2U1-AS1,RAP2C-AS1,SEPTIN7P6,ANKRD10-IT1,MIR600HG,LINC01139,LINC00923,C1orf220,HERC2P7,BTG3-AS1,GREB1L-DT,CCDC183-AS1,PKD1P4,LINC00265,LINC01881,LINC01358,DNMBP-AS1,ANAPC1P2,LRRFIP1P1,DNM1P46,LINC01016,PHKA2-AS1,PLCG1-AS1,ZNF812P,PDE4DIPP2,HERC2P3,CNTNAP3P2,BMS1P1,RALGAPA1P1,ADAMTS9-AS1,CERS6-AS1,LINC00106,PGM5P4,FAM153B,CLMAT3,GNRHR2,DLEU2L,PARGP1,MIR133A1HG,CBX3P2,TMC3-AS1,HNRNPA1P15,FAM185BP,ECI2-DT,CLRN1-AS1,COL6A4P1,DNM1P47,PKD1P6,RRM2P3,LRRC37A4P,SLC35E2A,TWF1P1,PIPSL,GUSBP2,KRTAP5-AS1,LNCOC1,LINC02078,LINC00639,TMED10P2,SMC2-DT,ADAMTS19-AS1,C5orf64,TTC3P1,ZNRF2P2,OR2A20P,AIDAP2,SMURF2P1,VWFP1,LINC01237,SMG1P3,ZNF658B,LMNTD2-AS1,HTATSF1P2,CSPG4P10,POM121B,CLIC4P1,PPM1K-DT,LINC01508,LINC02057,MSNP1,PCA3,LRRC37A6P,LINC02587,IGFL2-AS1,GNAQP1,VN1R83P,KCTD9P2,SIAH2-AS1,LINC02541,FAM106A,WARS2-IT1,PPIC-AS1,RPS3AP38,H2BP1,RERG-IT1,NPTN-IT1,LINC02296,GHET1,PKD1P3,PDE4DIPP5,KCTD9P4,PRICKLE2-AS1,GLIS2-AS1,FALEC,THAP12P7,CEP170P1,PHC1P1,FLNC-AS1,MRPS31P5,MYCBP2-AS1,LRRC37A16P,USP32P2,GPR199P,SVIL2P,DLX6-AS1,TPM1-AS,RORB-AS1,MEIS3P2,KCNMA1-AS1,LINC02388,MIR302CHG,CSPG4P13,TCAF1P1,UNQ6494,KIAA0087,LINC02367,CCDC144B |
