# Supplementary figures and images for: LncRNA GAS5 Regulates Myometrial Cell Contractions in an m6A-Dependent Manner
Source: Function (Oxf). 2025 Mar 7;6(2):zqaf009. doi: 10.1093/function/zqaf009 (PMC11931615; doi:10.1093/function/zqaf009)

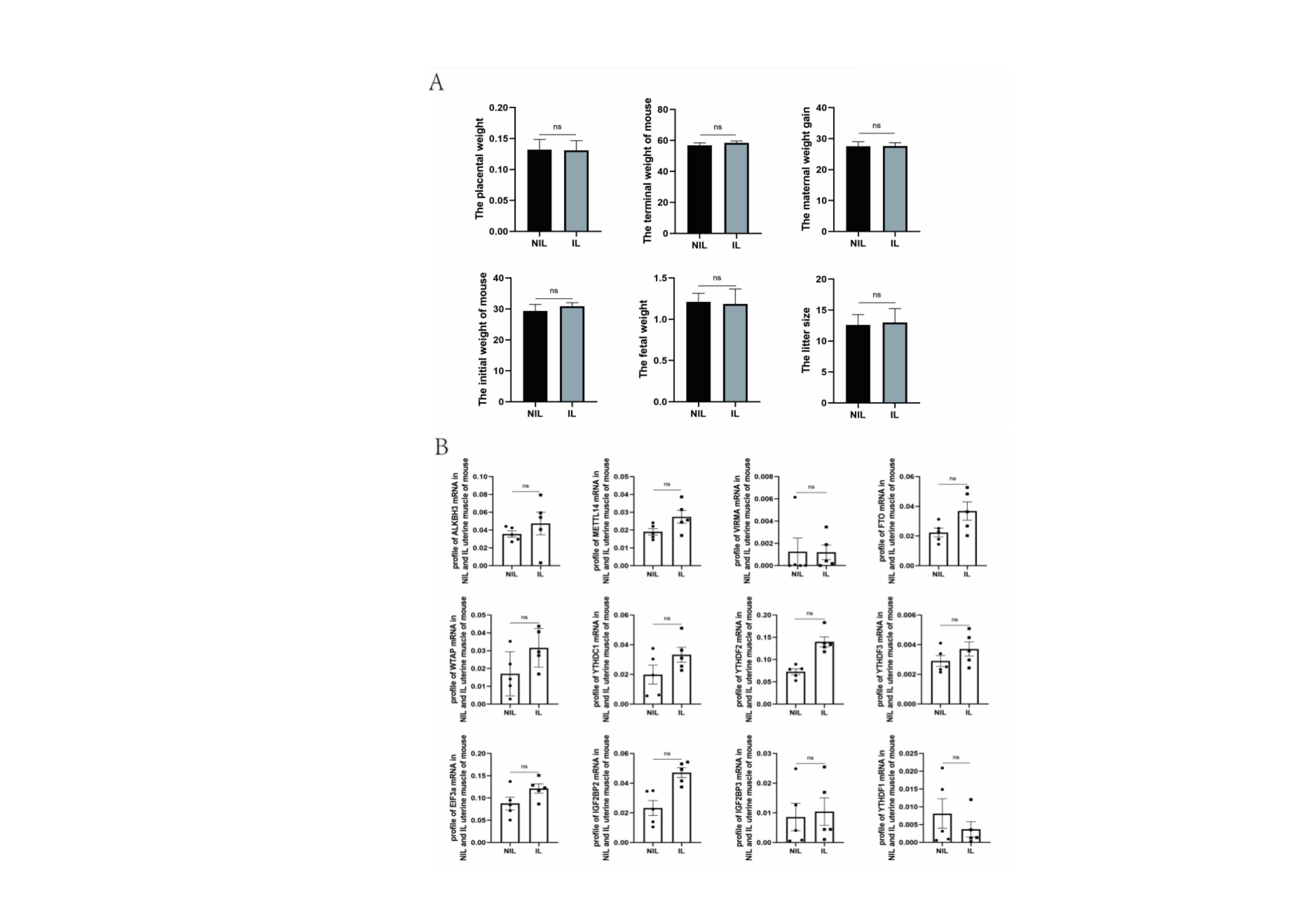

Supplement: zqaf009_Supplemental_Files [file zqaf009_supplemental_files.zip › Figure S1.jpg]

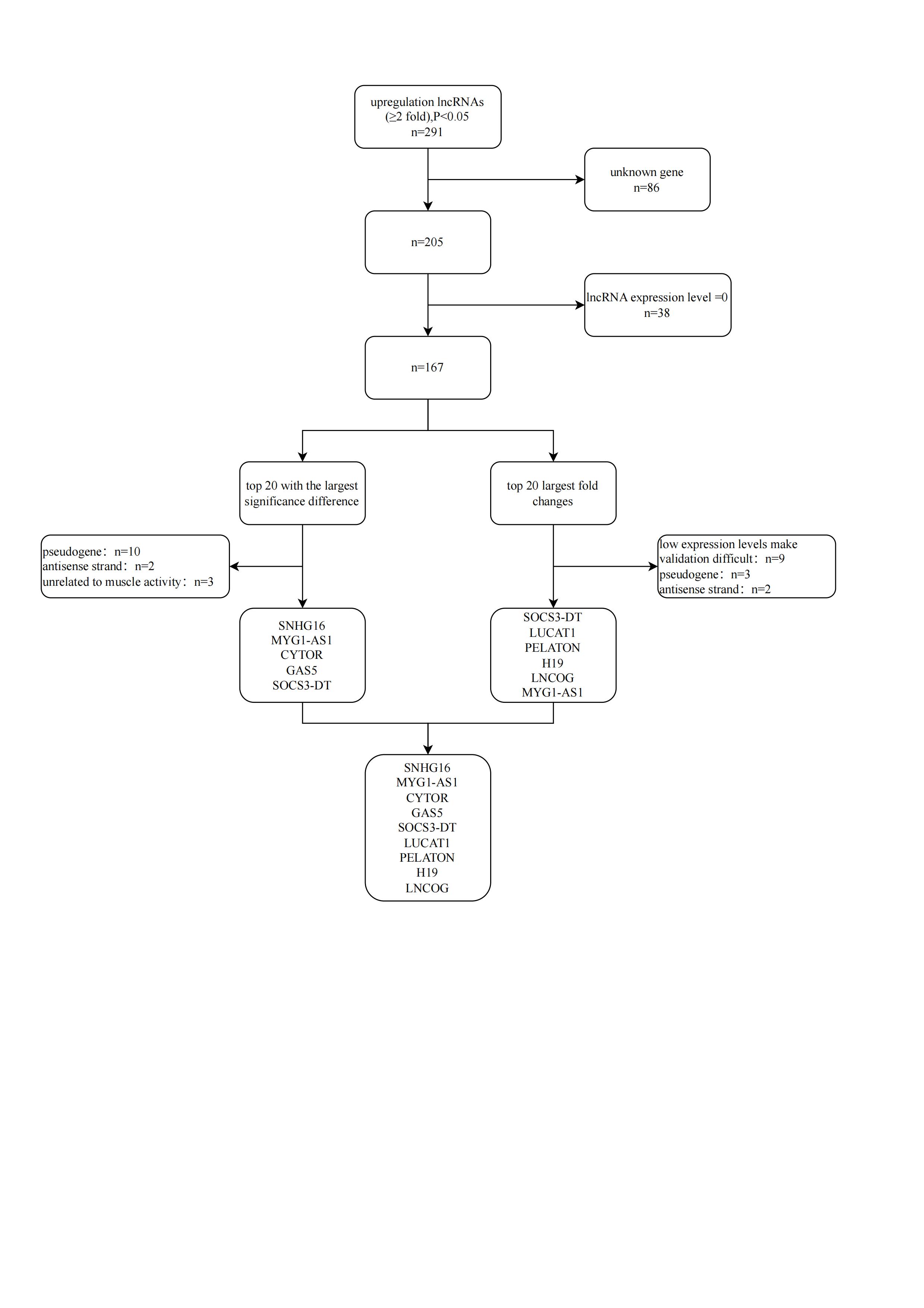

Supplement: zqaf009_Supplemental_Files [file zqaf009_supplemental_files.zip › Figure S2.jpg]
