# Supplementary material for: LncRNA GAS5 Regulates Myometrial Cell Contractions in an m6A-Dependent Manner
Source: Function (Oxf). 2025 Mar 7;6(2):zqaf009. doi: 10.1093/function/zqaf009 (PMC11931615; doi:10.1093/function/zqaf009)
Supplement: zqaf009_Supplemental_Files [file zqaf009_supplemental_files.zip › Table S1.doc]

Table S1 siRNA sequences

|  | Sense 5’ -3’ | antisense（5'-3'） |
| --- | --- | --- |
| IGF2BP1-1# | GGCUCAGUAUGGUACAGUA | UACUGUACCAUACUGAGCC |
| IGF2BP1-2# | AGCAAGAUACCGAGACAAA | UUUGUCUCGGUAUCUUGCU |
| IGF2BP1-3# | CGGUGAACGAGUUGCAGAA | UUCUGCAACUCGUUCACCG |
| METTL3-2# | CAGUGGAUCUGUUGUGAUA | UAUCACAACAGAUCCACUG |
| METTL3-3# | CAGACGAAUUAUCAAUAAA | UUUAUUGAUAAUUCGUCUG |
| METTL3-4# | GCAAGUAUGUUCACUAUGA | UCAUAGUGAACAUACUUGC |
| GAS5 166 | CAGAAAGCUGGAAGUUGAAAUGGUG | CACCAUUUCAACUUCCAGCUUUCUG |
| GAS5 242 | UAUCCAUGGAUGACUUGCUUGGGUA | UACCCAAGCAAGUCAUCCAUGGAUA |
| GAS5 351 | GCAAAGGACUCAGAAUUCAUGAUUG | CAAUCAUGAAUUCUGAGUCCUUUGC |
| TPM4-909 | GGAUCAGACACUAAACGAATT | UUCGUUUAGUGUCUGAUCCTT |
| TPM4-219 | GCACCUCCAGAAGAAACUATT | UAGUUUCUUCUGGAGGUGCTT |
| TPM4-516 | UCAGGAGAUGCAGCUCAAATT | UUUGAGCUGCAUCUCCUGATT |
